# Film dressings from Thai mango seed kernel extracts versus nanocrystalline silver dressings in antibacterial properties

**DOI:** 10.3389/jpps.2024.12674

**Published:** 2024-03-28

**Authors:** Sukhontha Hasatsri, Bajaree Jantrapanukorn

**Affiliations:** ^1^ Department of Pharmacy Practice, College of Pharmacy, Rangsit University, Pathum Thani, Thailand; ^2^ Faculty of Medical Technology, Rangsit University, Pathum Thani, Thailand

**Keywords:** antibacterial property, nanocrystalline silver dressing, film dressing, mango seed kernel, *Mangifera indica*, physical property, absorption property, water vapor transmission rate

## Abstract

**Introduction:** The extract from the Mango Seed Kernel (MSK) has been documented to exhibit antibacterial activity against Gram-positive and Gram-negative bacteria, including *Staphylococcus aureus* and *Pseudomonas aeruginosa*. This suggests that biomaterials containing MSK extract could be a viable alternative to conventional wound treatments, such as nanocrystalline silver dressings. Despite this potential, there is a notable gap in the literature regarding comparing the antibacterial effectiveness of MSK film dressings with nanocrystalline silver dressings. This study aimed to develop film dressings containing MSK extract and evaluate their antibacterial properties compared to nanocrystalline silver dressings. Additionally, the study aimed to assess other vital physical properties of these dressings critical for effective wound care.

**Materials and methods:** We prepared MSK film dressings from two cultivars of mango from Thailand, ‘Chokanan’ and ‘Namdokmai’. The inhibition-zone method was employed to determine the antibacterial property. The morphology and chemical characterization of the prepared MSK film dressings were examined with scanning electron microscopy (SEM) and Fourier-Transform Infrared Spectroscopy (FTIR), respectively. The absorption of pseudo-wound exudate and water vapor transmission rate (WVTR) of film dressings were evaluated.

**Results:** The results showed that 40% of MSKC film dressing had the highest inhibition zone (20.00 ± 0.00 mm against *S. aureus* and 17.00 ± 1.00 mm against *P. aeruginosa*) and 20%, 30%, and 40% of MSKC and MSKN film dressings had inhibition zones similar to nanocrystalline silver dressing for both *S. aureus* and *P. aeruginosa* (*p* > 0.05). In addition, all concentrations of the MSK film dressings had low absorption capacity, and Chokanan MSK (MSKC) film dressings had a higher WVTR than Namdokmai MSK (MSKN) film dressings.

**Conclusion:** 20%, 30%, and 40% of MSK film dressing is nearly as effective as nanocrystalline silver dressing. Therefore, it has the potential to be an alternative antibacterial dressing and is suitable for wounds with low exudate levels.

## Introduction

Gelatin is a natural polymer commonly used to develop wound dressing. However, it does not show any antibacterial effect. Gelfoam (Pfizer, United States of America) and Surgifoam (Ethicon, United States of America) are examples of wound dressings made from porcine gelatin [1]. Wound dressings composed of a combination of gelatin and plant extract to develop bioactive wound dressing exhibit several advantages, such as antibacterial properties [1]. The antibacterial properties of natural substances have led researchers to develop biomaterials for wound care. The Thai mango (*Mangifera indica* L.) also has antibacterial potential. Thai mango is one of the most popular fruits and is usually used to prepare various desserts. Mango consists of pulp, peel, and seed kernel [2]. Mango peel and seed kernel are commonly discarded as waste. Several studies have shown the bacterial activity of mango seed kernel (MSK) extracts in different mango cultivars [3–10]. A previous study by Prastiyanto et al. [4] reported that the ethanolic MSK extracts of Cengkir, Kopyor, Golek, Kweni, Avocado, Arumanis, Manalagi kernels had antibacterial activity against multi-drug resistant *Pseudomonas aeruginosa* (MDR *P. aeruginosa*) isolated from wounds. Deep and Bhattacharyya [9] studied the methanolic and aqueous MSK extracts of Langra, Sindhri, and Totapuri kernels, and the results showed antibacterial activity against *Escherichia coli* (*E. coli*). Methanolic MSK extract has been reported to show inhibition towards Methicillin-Resistant *Staphylococcus aureus* (MRSA), *E. coli*, *Vibrio vulnificus*, and *Enterococcus faecalis* [6, 10]. The MSK extract also had antibacterial activity against *Staphylococcus aureus* (*S. aureus*) and *P. aeruginosa* [3, 5, 7, 8]. *S. aureus* and *P. aeruginosa* are generally found in chronic wounds [11]. The top layer of wounds commonly detects *S. aureus*, whereas *P. aeruginosa* is often involved in infection of the deepest wound area [11]. Therefore, developing wound dressings containing MSK extract has the potential as a biomaterial for treating wound infection. However, no studies have reported the antibacterial effects of MSK film dressing compared with silver dressing, especially nanocrystalline silver products (Acticoat™). Nanocrystalline silver products are regularly used in wound care due to broad-spectrum antimicrobial activity but are typically more expensive than non-medicated wound dressings [12]. In contrast, MSK film dressings offer a more economically viable and environmentally sustainable alternative. Hence, this study was undertaken to develop MSK film dressings and evaluate their antibacterial properties compared to nanocrystalline silver dressing and other physical properties essential for wound care.

## Materials and methods

### Materials

Two Thai mango cultivars, ‘Chokanan’ and ‘Namdokmai’, were collected from Pathum Thani, Thailand. The limed bone gelatin with 250 bloom was purchased from Chemipan Corporation Co., Ltd. (Bangkok, Thailand). Nanocrystalline silver dressing (Acticoat) manufactured by Smith and Nephew Public Limited Company, London, United Kingdom.

### Process of MSK extraction

**FIGURE 1:**
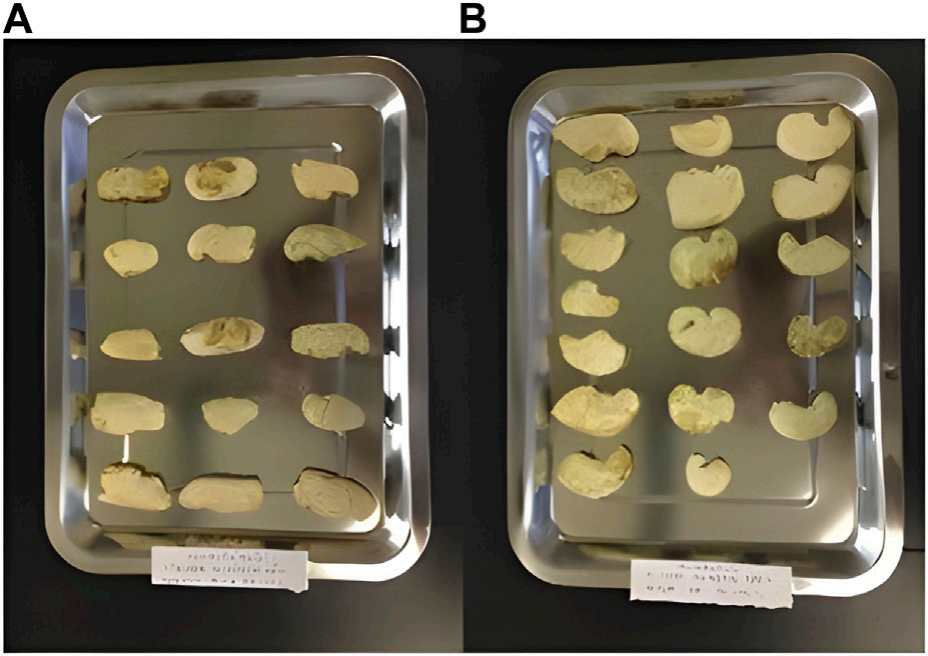
Mango seed kernels of **(A)** Chokanan (MSKC) and **(B)** Namdokmai (MSKN).

The mango seed kernels of Chokanan (MSKC) and Namdokmai (MSKN) were manually removed from the seeds and dried at 60°C for 24 h in a hot air oven (Memmert UFB 400, Memmert, Germany) (Figure 1). Then, the MSKC and MSKN were crushed using mortar and pestle to obtain MSK powder. The MSK solution (10%w/v) was prepared by dissolving the MSK powder in deionized water at room temperature and stirring continuously in the dark for 48 h at 125 rpm on a digital orbital shaker (DaiHan SHO-2D, Vietnam). The MSK solution was filtered through a filter fabric and centrifuged for 1 hour at 5,000 rpm (Hettich Centrifuge, Universal 320R, Germany). The supernatant of MSK extracts was collected and concentrated using a Rotary evaporator (Eyela N-1110V, Eyela, Japan) at 80°C until its volume decreased by 50%.

### Preparation of MSK film dressings

The gelatin solution (10%w/v) was prepared by dissolving dried gelatin in deionized water, and the solution was heated at 40°C for 2 hours. The MSK extracts/gelatin mixed solutions were prepared in five different ratios of MSK extracts to gelatin [40:60 (I), 30:70 (II), 20:80 (III), 10:90 (IV), 0:100 (V)] and dried at 25°C for 48 h to produce film dressings. The film dressings were sterilized with gamma rays at radiation doses of 10 kGy (adapted from Santos et al., 2011 [13]).

### Antibacterial properties

The antibacterial properties of the film dressing were evaluated using the inhibition-zone method following the procedures recommended by the Clinical and Laboratory Standards Institute (CLSI) and performed under a laminar flow cabinet [14]. *S. aureus* and *P. aeruginosa*, representing Gram-positive and Gram-negative bacteria, respectively, were chosen because they are common wound pathogens. The bacterial cultures were diluted to a final concentration of 1 × 10^8^ CFU/mL, and this diluted solution was then spread on agar plates. Then, the film dressing (1.5 cm × 1.5 cm) was placed on each plate and incubated at 37°C for 22 h. After incubation, the diameters of inhibition zones were measured. Gelatin film dressing was included as a negative control, and the gentamicin disc (10 µg/disc) and nanocrystalline silver dressing (Acticoat, Smith and Nephew Public Limited Company, United Kingdom) were included as a positive control. The MSK film dressings were prepared in four concentrations: 10%, 20%, 30% and 40%. The experiments were performed in triplicate.

### Morphology characterization

In the initial step, film dressing was prepared by attaching it to the aluminium stubs and then coating it with gold. The Scanning Electron Microscope (SEM, JSM-IT300, JEOL, Japan) was used to analyze the morphology of the MSK film dressing with the surface (700x) and cross-sectional (700x) images. The observation was performed at 15 kV.

### Chemical characterization

The film dressing (1 cm × 1 cm) was prepared. The Fourier Transform Infrared Spectroscopy (FTIR) spectra were recorded using a Spectrum GX FTIR spectrometer (PerkinElmer, United States of America) in the 650–4,000 cm^−1^ range.

### Absorption properties

The absorption properties of the film dressing were evaluated using BS EN 13726-1: 2002, Test methods for primary wound dressing, Part 1, specifically *Morphology characterization* section: free swell absorptive capacities with slight modifications [15]. BS EN 13726 is the international standard for conducting laboratory tests on wound dressings. The film dressing (2 cm × 2 cm) was prepared, and its weight was recorded. A test solution was prepared by dissolving 8.298 g of NaCl (0.142 mol/L) and 0.367 g of CaCl_2_2H_2_O (0.0025 mol/L) in 1 L of deionized water. The film dressing was submerged in the test solution and incubated at 37°C. The dressing was weighed at various time intervals (15 min, 30 min, 1 h, 2 h, 4 h, and 6 h). The experiments were performed in triplicate.

### Water vapor transmission rate

The film dressing’s water vapor transmission rate (WVTR) was examined using BS EN 13726-2:2002, Test methods for primary wound dressing, Part 2 moisture vapor transmission rate of permeable film dressings with slight modifications [16]. A bottle (diameter 1.2 cm) of the test solution was covered with the film dressing and incubated at 37°C. The dressing was weighed at various time intervals (15 min, 30 min, 1 h, 2 h, 4 h, and 6 h). The experiments were performed in triplicate.

### Statistical analysis

Data were expressed as mean ± standard deviation. The results were statistically analyzed using one-way analysis of variance (ANOVA) followed by *post hoc* tests with IBM SPSS Statistics version 22 software (SPSS Inc., Chicago, United States of America), and *p* < 0.05 was considered to be statistically significant.

## Results

### Antibacterial properties

This study evaluated the antibacterial activity of MSK film dressing against *S. aureus* and *P. aeruginosa* using the inhibition-zone method. All MSK film dressings consist of MSK extracts and gelatin. Gelatin film dressing is a negative control. Nanocrystalline silver dressing and gentamicin disc are positive controls. The inhibition zones (mm) of the film dressings are shown in Table 1. The images of the antibacterial properties of the film dressings are shown in Supplementary Material. Gentamicin discs (10 µg/disc) showed the highest antibacterial activity (26.33 ± 0.58 mm and 18.50 ± 0.50 mm for *S. aureus* and *P. aeruginosa*, respectively). In comparison, gelatin film dressing did not have antibacterial activity. All the MSK film dressings from aqueous MSK extract exhibited inhibition against *S. aureus* and *P. aeruginosa* and showed dose-dependent antibacterial activity. The highest antibacterial activity of MSK film dressing was demonstrated in 40% of mango seed kernels of MSKC film dressing (20.00 ± 0.00 mm and 17.00 ± 1.00 mm for *S. aureus* and *P. aeruginosa*, respectively). Moreover, when comparing MSK film dressings of both Thai mango cultivars (Chokanan and Namdokmai) with commercial wound dressings (nanocrystalline silver dressing), it was found that 20%, 30%, and 40% of MSKC film dressings and all concentrations of mango seed kernels of MSKN film dressings had inhibition zone similar to nanocrystalline silver dressing for *S. aureus* (*p* > 0.05). All concentrations of mango seed kernels of MSKC and MSKN film dressings had inhibition zones similar to nanocrystalline silver dressing for *P. aeruginosa* (*p* > 0.05). In addition, it was found that 40% of MSKC and MSKN film dressings had inhibition zones similar to gentamicin for *P. aeruginosa* (*p* > 0.05).

**TABLE 1:** Inhibition zone (mm) of dressings.

Film dressings	Inhibition zone (mm)	
	*S. aureus*	*P. aeruginosa*
Gelatin (negative control)	0.00^a^ ^*^ ^b^ ^*^	0.00^a^ ^*^ ^b^ ^*^
10% MSKC	13.33 ± 0.58^a^ ^*^ ^b^ ^*^ ^c^ ^*^	15.67 ± 1.15^b^ ^*^ ^c^ ^*^
20% MSKC	16.33 ± 0.58^b^ ^*^ ^c^ ^*^	15.67 ± 0.58^b^ ^*^ ^c^ ^*^
30% MSKC	17.67 ± 2.31^b^ ^*^ ^c^ ^*^	15.33 ± 0.58^b^ ^*^ ^c^ ^*^
40% MSKC	20.00 ± 0.00^b^ ^*^ ^c^ ^*^	17.00 ± 1.00^c^ ^*^
10% MSKN	18.33 ± 0.58^b^ ^*^ ^c^ ^*^	15.67 ± 0.58^b^ ^*^ ^c^ ^*^
20% MSKN	18.00 ± 1.00^b^ ^*^ ^c^ ^*^	15.33 ± 0.58^b^ ^*^ ^c^ ^*^
30% MSKN	19.00 ± 0.00^b^ ^*^ ^c^ ^*^	15.33 ± 0.58^b^ ^*^ ^c^ ^*^
40% MSKN	19.33 ± 0.58^b^ ^*^ ^c^ ^*^	16.33 ± 0.58^c^ ^*^
Nanocrystalline silver dressing (positive control)	18.00 ± 1.00^b^ ^*^ ^c^ ^*^	17.00 ± 1.00^c^ ^*^
Gentamicin disc (positive control)	26.33 ± 0.58^a^ ^*^ ^c^ ^*^	18.50 ± 0.50^c^ ^*^

Data were expressed as mean ± standard deviation.

^a^ Compared to the nanocrystalline silver dressing.

^b^ Compared to the gentamicin disc.

^c^ Compared to the gelatin.

*p < 0.05; MSKC, mango seed kernels of Chokanan; MSKN, mango seed kernels of Namdokmai.

### Morphology characterization

**FIGURE 2:**
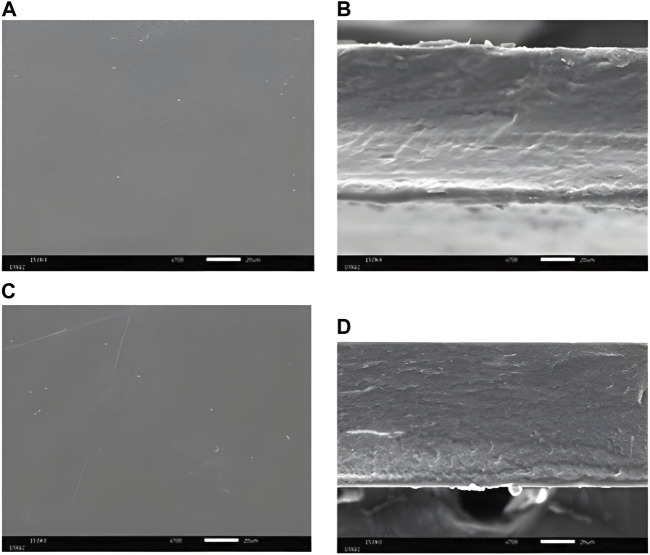
SEM photograph of **(A)** the surface, **(B)** the cross-section of 40% MSKC film dressing, **(C)** the surface, and **(D)** the cross-section of 40% MSKN film dressing.

The surface and cross-section of the 40% MSK film dressings are shown in Figure 2. Supplementary Material shows all the surface and cross-sections of the MSK and gelatin film dressings. All film dressings presented smooth surfaces and were non-porous structures. The smooth surfaces of the films indicated that MSK distribution in the film is homogenous.

### Chemical characterization

**FIGURE 3:**
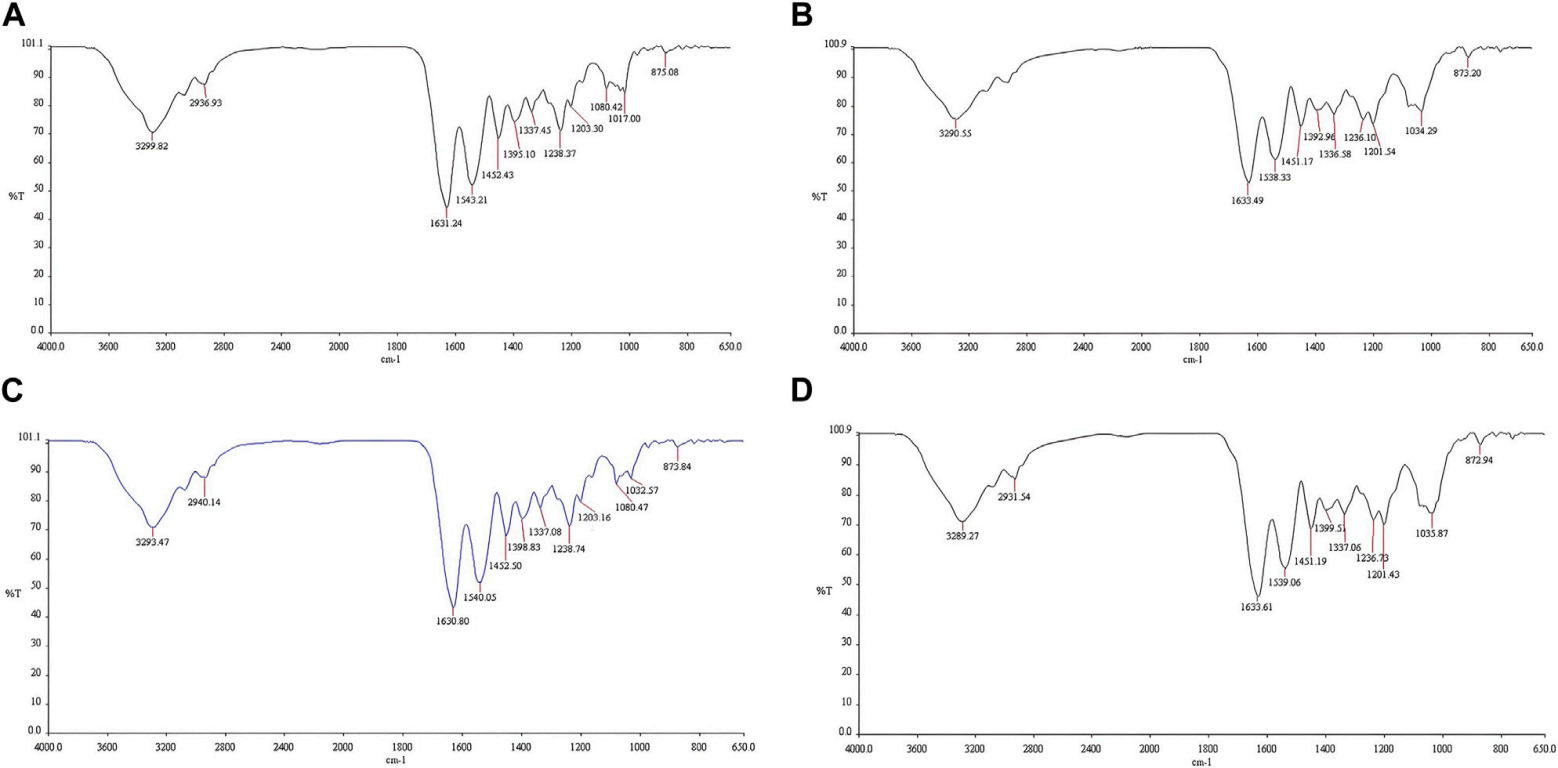
FTIR spectra of the **(A)** 10% MSKC film dressing, **(B)** 40% MSKC film dressing, **(C)** 10% MSKN film dressing, and **(D)** 40% MSKN film dressing.

The FTIR spectra of the 10% and 40% MSK are shown in Figure 3. Supplementary Material shows all the FTIR spectra of the MSK and gelatin film dressings. Strong absorption bands appear at 1,630–1,634 cm^−1^ and 1,538–1,545 cm^−1^ due to the C=O and N-H/C-N bands in the gelatin [17, 18]. In addition, a strong absorption band appears at 1,032–1,036 cm^−1^ due to the C-O-H band in the MSK [18].

### Absorption properties

**FIGURE 4:**
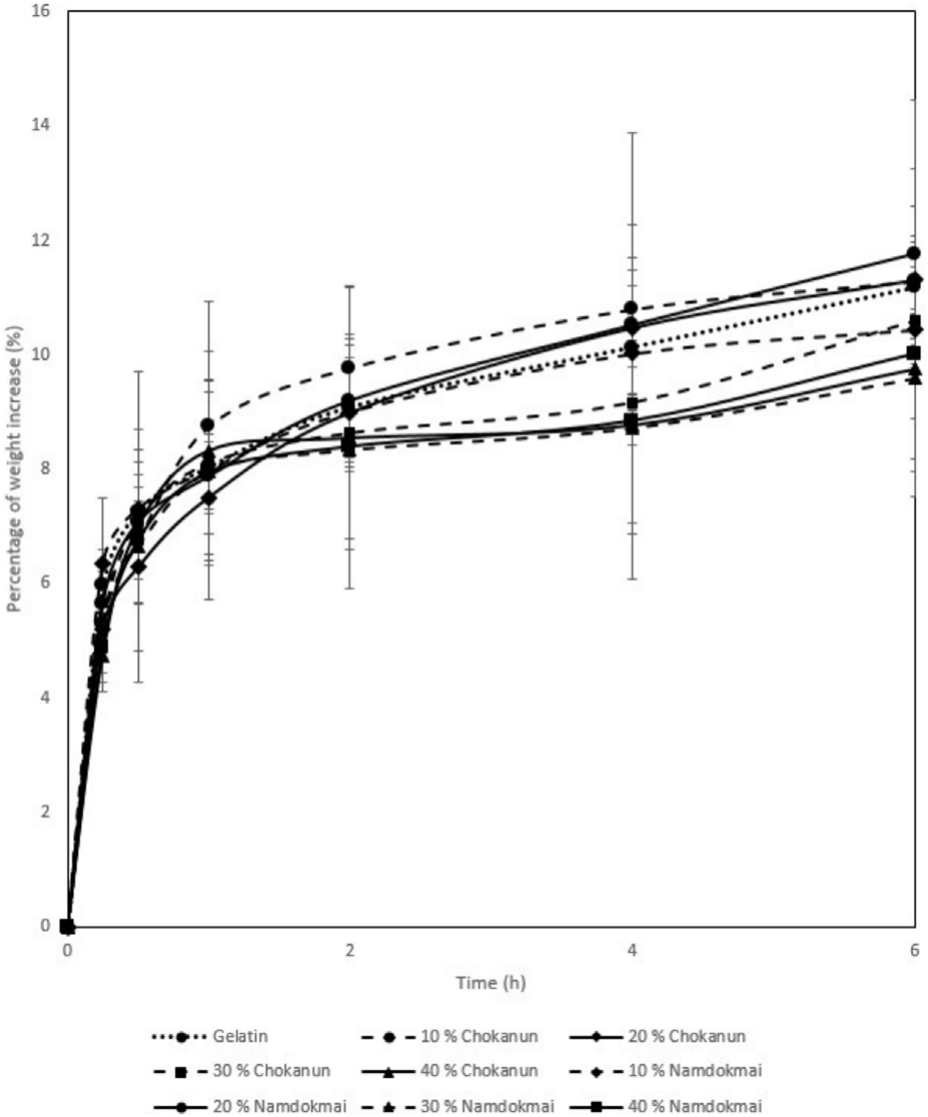
Absorption properties.

The absorption properties of the film dressings are shown in Figure 4. The results show that 10% and 20% of MSKC and MSKN film dressings had absorption capacity similar to gelatin film dressing. In contrast, 30% and 40% of MSKC and MSKN film dressings had lower absorption capacity than others. The higher MSK extract presented a lower absorption capacity.

### Water vapor transmission rate

**FIGURE 5:**
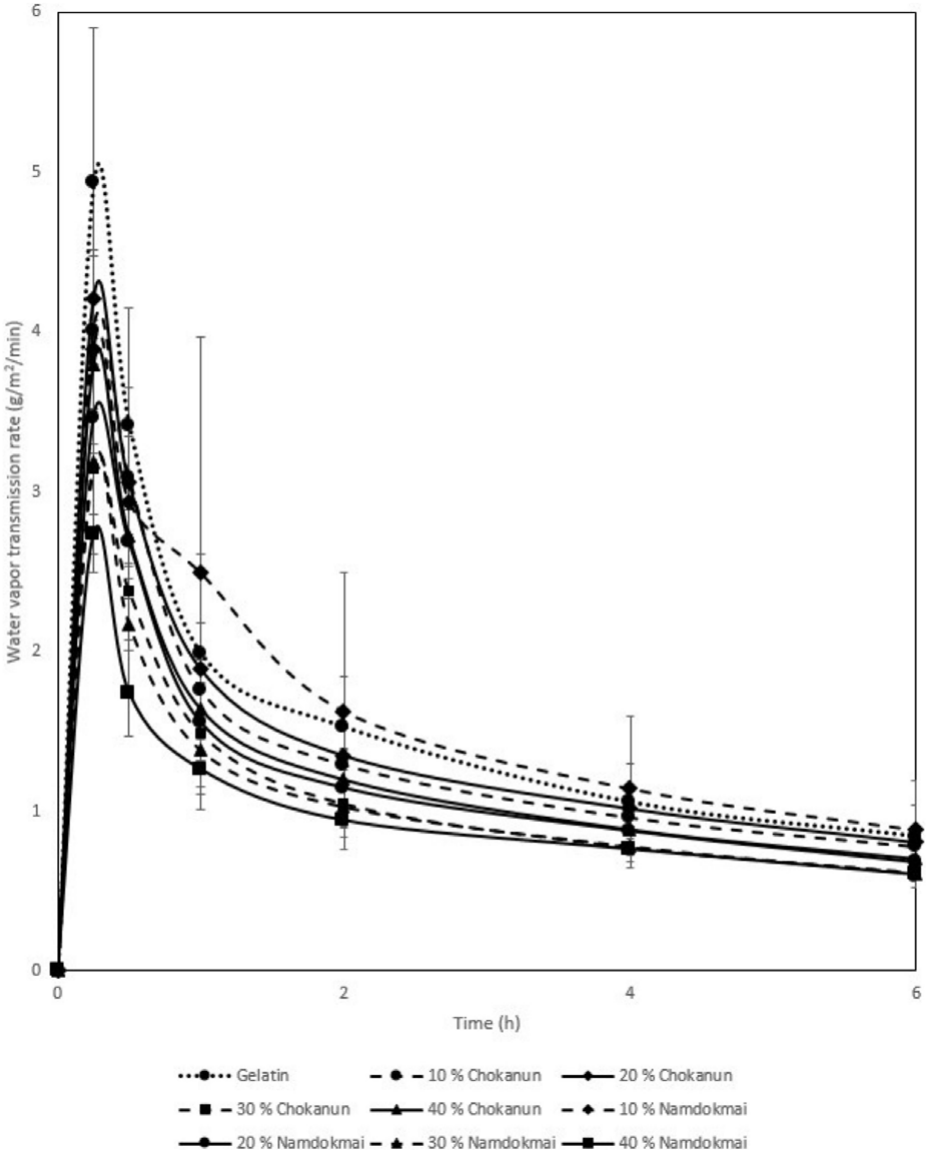
Water vapor transmission rate.

The WVTR properties of the film dressings are shown in Figure 5. All MSK film dressings had a lower WVTR than gelatin film dressing, and MSKC film dressings had a higher WVTR than MSKN film dressings. WVTR of all MSK film dressings decreased with increasing MSK concentration.

## Discussion

Mango Seed Kernel (MSK) extract has shown potential antibacterial properties against Gram-positive and Gram-negative bacteria causing wound infections. The ethanolic extracts of MSK from different mango cultivars originating in Indonesia exhibited antimicrobial activity against MDR *P. aeruginosa* isolated from wounds [4]. The ethanolic extracts of MSK from Thai mango (Fahlun, Namdokmai, Raet, Kaeo, and Kaeo Sawoei) showed antibacterial activity against *S. aureus* and *E. coli* [19, 20]. Furthermore, the aqueous and alcoholic MSK extracts of waterlily, lemak, and shakran exhibited antibacterial activity against *S. aureus*, *E. coli,* and *P. aeruginosa* [8]. Other research studies similarly indicated that the MSK extract exhibited antibacterial properties against *S. aureus* and *P. aeruginosa* [3, 5, 7, 8]. Apart from the antibacterial properties of MSK extracts, Espinosa-Espinosa et al. developed MSK ointment from Ataulfo kernel, and the result showed antibacterial activity against *S. aureus* and *P. aeruginosa* [21].

In our study, we developed film dressings from two cultivars of Thai MSK extract. Film dressings offer the benefit of creating a protective barrier against microorganism contamination, protecting the wound from mechanical trauma, and allowing for gaseous exchange [22–24]. Nevertheless, currently, there is a lack of research exploring the development of film dressings derived from MSK extracts and studies about absorption properties and WVTR to provide gaseous exchange characteristics. In clinical practice, nanocrystalline silver dressing with bactericidal action is commonly utilized for managing infected wounds [25]. Thus, the MSK film dressing should be compared with the nanocrystalline silver dressing to provide antibacterial data for better clinical decision-making. Considering all the reasons mentioned above, this study aims to develop film dressing containing MSK extract as an alternative to compare with nanocrystalline silver dressing.

The results of morphology characterization showed that our MSK film dressings were non-porous structures, which is a characteristic of film dressings [26]. Similar observations were reported for corn starch and glycerol film dressings [27].

In addition, the results of chemical characterization found that there were decreased intensities in the absorption band of C=O (1,630-1,634 cm^−1^) and N-H/C-N (1,538–1,545 cm^−1^) in the MSK film dressings due to lower gelatin concentrations [17]. In contrast, increased intensities in the absorption band of C-O-H (1,032–1,036 cm^−1^) in the MSK film dressings due to higher MSK concentrations [18].

Regarding the antibacterial efficacy, the study revealed that 20%, 30%, and 40% of MSKC and MSKN film dressings had inhibition zones similar to nanocrystalline silver dressing for both *S. aureus* and *P. aeruginosa* (*p* > 0.05). Additionally, 40% of MSKC and MSKN film dressings had inhibition zones similar to control antibiotics for *P. aeruginosa* (*p* > 0.05).

In the prior study, the inhibition zone of aqueous MSK extract of Waterlily, Lemak, and Shakran against *S. aureus* and *P. aeruginosa* was determined by Abdullah et al. [8], using a higher concentration of MSK extracts and different form compared to our study. The diameter of the inhibition zone for *S. aureus* was similar to our study [8]. However, the diameter of the inhibition zone for *P. aeruginosa* was lower than in our study [8]. Another study reported that Thai MSK extract from Fahlun at a concentration of 0.625, 1.25, 2.5, and 5 mg/disc had an inhibition zone of 11.44 ± 0.59, 14.81 ± 0.49, 13.94 ± 1.34, and 17.06 ± 3.23 mm, respectively against *S. aureus* [19]. In this study, the MSK film dressings had similar antibacterial activity to the Thai MSK extract from Fahlun (5 mg/disc) against *S. aureus* [19]. The results of the antibacterial efficacy showed that film dressings impregnated with MSK extract from two cultivars of Thai mango (Chokanan and Namdokmai) had the potential as an alternative to nanocrystalline silver dressing with comparable antibacterial efficacy, especially 40% of MSK film dressing. The disadvantage of nanocrystalline silver dressing is cytotoxicity against keratinocytes and fibroblasts, leading to delayed wound healing [28]. It has been reported that the ability of MSK extract to inhibit the growth of bacteria relates to flavonoids and tannins [4, 9, 29, 30]. The flavonoid can damage bacterial cell walls, resulting in bacterial death [31]. In addition, previous study strongly supports the antibacterial activity of tannins extracted from MSK against *S. aureus* and *E. coli* [30].

Furthermore, we found that the antibacterial activity of MSK film dressings was more effective against Gram-positive than Gram-negative. Mirghani et al. [5] also showed differences in the antibacterial activity of ethanolic MSK extract of Blackgold, Waterlily, and Lemak kernels between Gram-positive and Gram-negative bacteria. The variations in the antibacterial activity are because of lipopolysaccharides in the Gram-negative bacteria’s outer membrane [32]. Gram-negative bacteria’s outer membrane is the primary cause of their ability to resist a broad spectrum of antibiotics [33].

However, apart from their antibacterial properties, the physical properties of wound dressings are also crucial. In this study, all MSK film dressings demonstrated a little absorption capacity. Gamma irradiation is the reason for the reduced absorption capacity [34]. This study used a rotary evaporator to remove solvent from MSK extracts, and then the MSK film dressings were sterilized with gamma rays. The absorption capacity of the film dressings was affected by the gamma irradiation due to the degradation of peptide chains of the gelatin [34]. In addition, all MSK film dressings had a lower absorption capacity than other dressing types because of their structure [35]. A porous or fibrous structure can absorb fluids well [35, 36]. The SEM photograph of a cross-section of film dressings (see Figure 2) showed that they were non-porous structures. Therefore, the MSK film dressing suits wounds with low exudate levels.

The WVTR of a wound dressing is also essential because it directly controls the moisture environment [37]. However, if the MSK film dressing is used in a wound with low exudate levels, WVTR should not be high to avoid wound desiccation (dry wounds) or dressing adherence. A dry wound environment delays autolytic debridement, angiogenesis, formation of granulation tissue, and keratinocyte migration [38]. Our results showed that the WVTR of MSK film dressings was low. Therefore, the MSK film dressing had appropriate WVTR for wounds with low exudate levels.

## Conclusion

Compared to nanocrystalline silver dressings, this study is the first to provide the antibacterial properties of MSK film dressings from Chokanan and Namdokmai. The highest antibacterial activity of MSK film dressing against *S. aureus* and *P. aeruginosa* showed in 40% of MSKC film dressing. Additionally, 20%, 30%, and 40% of MSKC and MSKN film dressings had inhibition zones similar to nanocrystalline silver dressings for both *S. aureus* and *P. aeruginosa*. The findings from absorption properties and WVTR tests suggest that the MSK film dressing is appropriate for wounds with low exudate levels. Further research is needed to ensure that the MSK film dressing is suitable for wound infection treatment in an *in vivo* model.

## Data Availability

The original contributions presented in the study are included in the article/Supplementary Material, further inquiries can be directed to the corresponding author.
