# Supplementary material for: Film dressings from Thai mango seed kernel extracts versus nanocrystalline silver dressings in antibacterial properties
Source: J Pharm Pharm Sci. 2024 Mar 28;27:12674. doi: 10.3389/jpps.2024.12674 (PMC11006813; doi:10.3389/jpps.2024.12674)
Supplement: Supplementary file 1 [file DataSheet1.docx]

Supplementary Material

# Antibacterial properties


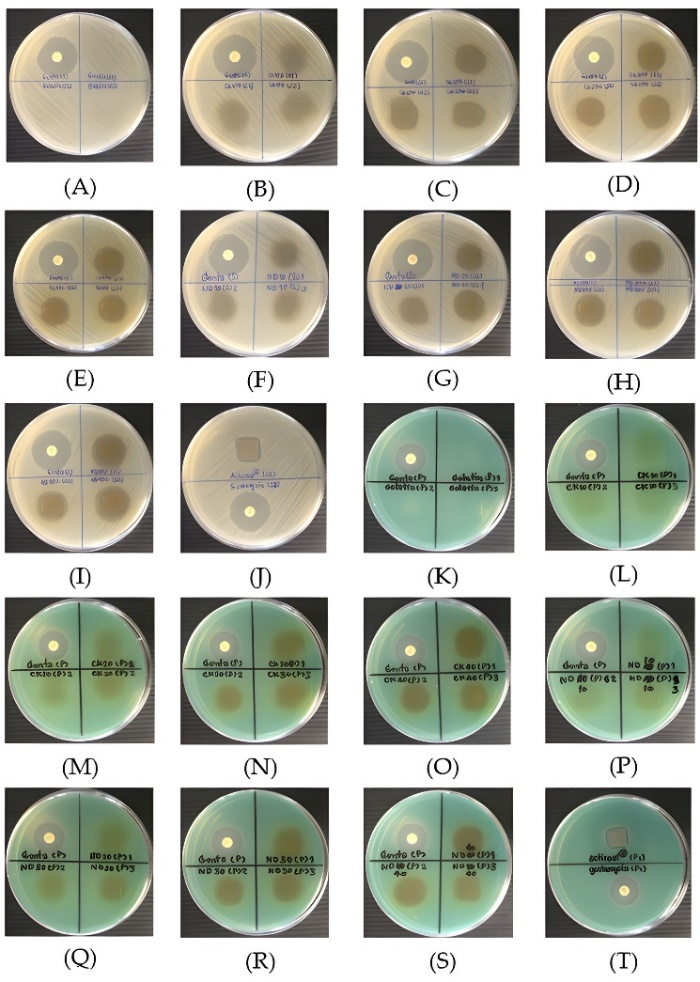


**Figure 1.** Antibacterial properties of (a) a gelatin film dressing, (b) 10% MSKC film dressing, (c) 20% MSKC film dressing, (d) 30% MSKC film dressing, (e) 40% MSKC film dressing, (f) 10% MSKN film dressing, (g) 20% MSKN film dressing, (h) 30% MSKN film dressing, (i) 40% MSKN film dressing, (j) nanocrystalline silver dressing against *S. aureus* ; (k) a gelatin film dressing, (l) 10% MSKC film dressing, (m) 20% MSKC film dressing, (n) 30% MSKC film dressing, (o) 40% MSKC film dressing, (p) 10% MSKN film dressing, (q) 20% MSKN film dressing, (r) 30% MSKN film dressing, (s) 40% MSKN film dressing, (t) nanocrystalline silver dressing against *P. aeruginosa*.

# SEM Photograph


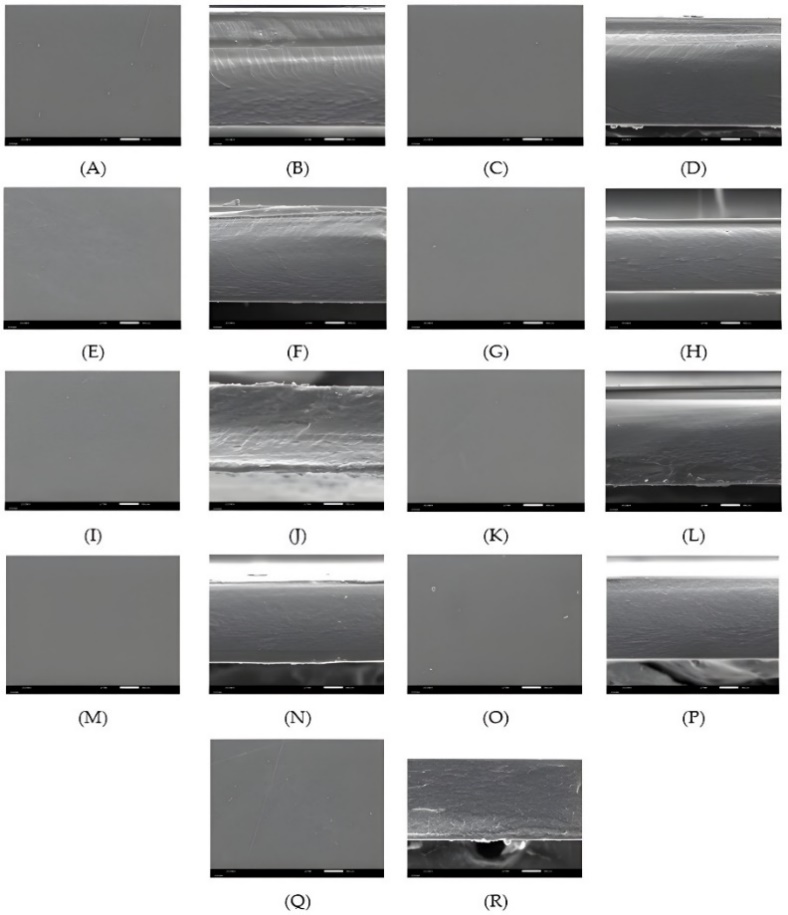


**Figure 2.** SEM photograph of (a) the surface, (b) cross-section of a gelatin film dressing; (c) the surface, (d) cross-section of 10% MSKC film dressing; (e) the surface, (f) cross-section of 20% MSKC film dressing; (g) the surface, (h) cross-section of 30% MSKC film dressing; (i) the surface, (j) cross-section of 40% MSKC film dressing; (k) the surface, (l) cross-section of 10% MSKN film dressing; (m) the surface, (n) cross-section of 20% MSKN film dressing; (o) the surface, (p) cross-section of 30% MSKN film dressing; (q) the surface, (r) cross-section of 40% MSKN film dressing.

# FTIR Spectra


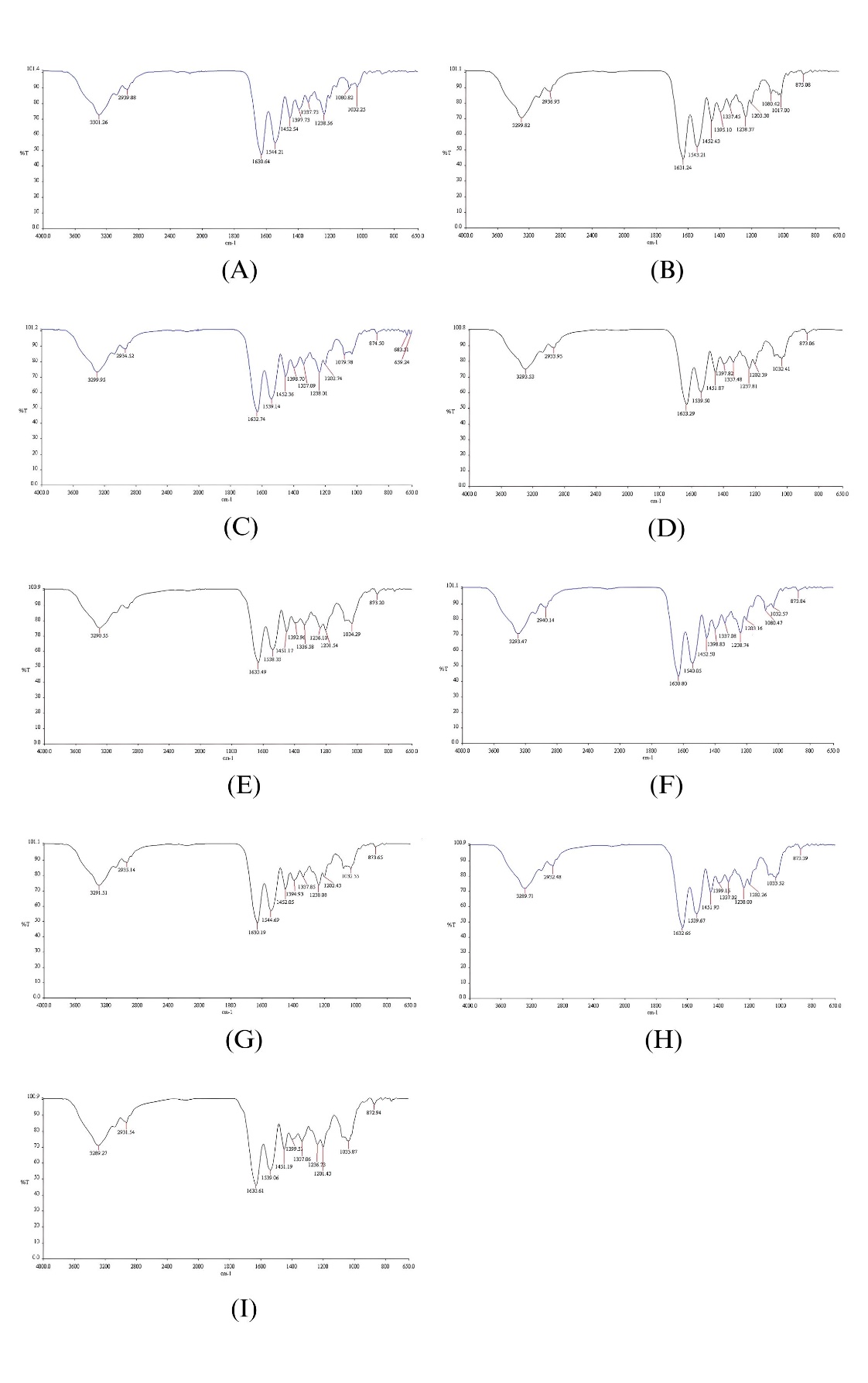


**Figure 3.** FTIR spectra of the (a) gelatin film dressing; (b) 10% MSKC film dressing; (c) 20% MSKC film dressing; (d) 30% MSKC film dressing; (e) 40% MSKC film dressing; (f) 10% MSKN film dressing; (g) 20% MSKN film dressing; (h) 30% MSKN film dressing; (i) 40% MSKN film dressing.
